# A methodological review of acupuncture for chronic atrophic gastritis: toward a core outcome set

**DOI:** 10.3389/fmed.2026.1818918

**Published:** 2026-06-01

**Authors:** Han Zhang, Yang Yu, Siyi Zheng, Shuaiyu Ying, Ding Shen, Wei Ye, Yichun Shi, Chenben Zhang, Xiaojing Wang, Yi Liang

**Affiliations:** 1 The Third School of Clinical Medicine, Zhejiang Chinese Medical University, Hangzhou, China; 2 Department of Massage, Hangzhou TCM Hospital of Zhejiang Chinese Medical University (Hangzhou Hospital of Traditional Chinese Medicine), Hangzhou, China; 3 Department of Gastroenterology, Hangzhou TCM Hospital of Zhejiang Chinese Medical University (Hangzhou Hospital of Traditional Chinese Medicine), Hangzhou, China; 4 Department of Gastroenterology, The Third Affiliated Hospital of Zhejiang Chinese Medical University (Zhongshan Hospital of Zhejiang Province), Hangzhou, China; 5 Department of Acupuncture and Moxibustion, The Third Affiliated Hospital of Zhejiang Chinese Medical University (Zhongshan Hospital of Zhejiang Province), Hangzhou, China

**Keywords:** acupuncture, chronic atrophic gastritis, core outcome set, outcome measures, PICOS, randomized controlled trial

## Abstract

**Introduction:**

This study systematically reviewed randomized controlled trials (RCTs) of acupuncture for chronic atrophic gastritis (CAG) published over the past 25 years, aiming to analyze the current status of clinical research, with a focus on evaluating heterogeneity in outcome measures and methodological quality.

**Methods:**

A comprehensive search of major Chinese and English databases identified 55 RCTs involving 5,311 patients. Methodological quality was assessed using the Cochrane risk of bias tool. Outcome measures were categorized and analyzed by frequency.

**Results:**

The results revealed notable methodological limitations in the current literature. Only 83.6% of the studies employed low-risk randomization methods, while very few implemented allocation concealment (3.6%) or blinding (1.8%). Furthermore, no study reported prospective registration, and 34.5% did not mention ethical approval. Regarding outcome measures, 57 different indicators were reported, with laboratory and imaging examinations accounting for the majority (55.4%). Notably, there was a severe lack of focus on patient-reported quality of life, health economic evaluation, and long-term disease prognosis. Considerable heterogeneity was observed in the naming, definition, and timing of outcome measurements, and most studies failed to clearly distinguish between primary and secondary outcomes.

**Discussion:**

In conclusion, the absence of clear criteria hinders clinical translation and results in fragmented data in existing clinical studies on acupuncture treatment for CAG. Standardized clinical trial protocols and a core outcome set (COS) for this discipline are urgently needed. Building on this foundation, future research should conduct multicenter, large-sample, methodologically robust RCTs with long-term follow-up. Such efforts will scientifically advance the standardized development of acupuncture clinical practice and produce high-quality, generalizable, evidence-based conclusions.

## 1 Introduction

Chronic atrophic gastritis (CAG) represents a subtype of chronic gastritis and is frequently recognized as a precancerous condition in clinical gastroenterology. The condition is defined as a chronic gastric disorder characterized by a reduction of gastric mucosal glands due to repeated epithelial damage, with or without intestinal metaplasia and pseudopyloric metaplasia. The primary causes include chronic Helicobacter pylori infection, which is the most prevalent etiology, and autoimmune disease (1–3). The incidence is high, with pathological tissue biopsy diagnoses of CAG constituting up to 25.8% of chronic gastritis patients in China. Prevalence and detection rates rise with increasing age (4). The gastric cancer progression model proposed by Correa and Piazuelo (5) delineates the sequence: normal gastric mucosa → superficial gastritis → chronic atrophic gastritis (CAG) → intestinal metaplasia → dysplasia → gastric cancer, highlighting the critical role of CAG in the transition from gastritis to cancer. Gastric mucosal dysplasia, also known as intraepithelial neoplasia, is widely acknowledged as a precancerous lesion associated with gastric cancer. Timely diagnosis and intervention for CAG and precancerous lesions of gastric cancer (PLGC) are essential for the prevention of gastric cancer. Western medical treatment primarily emphasizes the identification of underlying causes and the provision of symptomatic relief, which entails both benefits and drawbacks. Prolonged use, despite its effectiveness, is associated with potential adverse risks (6). Recent studies indicate that acupuncture has beneficial therapeutic effects in treating gastrointestinal disorders, while avoiding the adverse effects commonly associated with pharmaceutical interventions. This approach serves as a notable complementary therapy for CAG (7), necessitating additional research and advancement. In randomized controlled trials, which are the accepted standard for assessing clinical efficacy, the careful selection of outcome measures is a crucial factor in enhancing study design. This establishes the basis for bias control and research quality while also directly influencing the clinical applicability and translational value of study findings (8, 9). The frequency of clinical studies on CAG has progressively risen in recent years. In the domain of RCTs examining acupuncture treatment for CAG, there is an absence of standardized guidance regarding the selection of outcome measures. The existing literature demonstrates considerable variability in the quality of clinical evidence, primarily due to significant heterogeneity and a lack of standardization in outcome measure establishment. This significantly impedes the production of high-quality evidence and its application in clinical practice. This paper systematically analyzes the selection of outcome measures in RCTs of acupuncture for CAG over the past 25 years. This work emphasizes essential aspects of clinical trial design to offer methodological insights for the development of future RCT protocols, specifically concerning the scientific selection of outcome measures. It aims to establish a foundational framework for the gradual identification of a core set of outcome measures in acupuncture treatment for CAG.

## 2 Methods

### 2.1 Inclusion criteria

Studies that met the specified criteria were included. 1. Study type: Randomized controlled trials published in Chinese or English from 2000 to 2025. Chinese-language literature was limited to articles published in journals indexed by the Chinese Science Citation Database (CSCD), Chinese Core Journals, or the Chinese Science and Technology Papers Statistics Source (CSTPCD). In cases of duplicated studies, the selection was limited to the most comprehensive and recent publication. The blinding status was not significant. Language limitations: Only Chinese and English are permitted. 2. Study Population: Patients with a confirmed diagnosis of CAG, adhering to Western medical diagnostic criteria, with no restrictions on gender, age, ethnicity, or etiology of the disease. 3. Intervention measures: The experimental group utilized acupuncture-based therapies, which encompassed conventional filiform needle acupuncture, fire needle therapy, electroacupuncture, acupoint injection, acupoint thread implantation, and warm needle acupuncture. Acupuncture techniques, insertion depth, and retention time were not constrained. The control group was administered non-acupuncture interventions. Symptomatic medications utilized in both groups must be consistent.

### 2.2 Exclusion criteria

Exclude studies that fulfill the following criteria: 1. Participants including pregnant women, lactating women, or individuals with significant cardiovascular, cerebrovascular, hepatic, renal, or endocrine disorders or impairments of the hematopoietic system; those unable to adhere to examinations or treatment; individuals with a pathological diagnosis of high-grade intraepithelial neoplasia (severe dysplasia) or other concurrent diseases; 2. Patients were enrolled utilizing pseudo-randomization methods, specifically through “patient presentation order.” 3. Research that presents solely mechanism-related outcome measures (e.g., pathways, factors) without including clinical efficacy-related endpoints; 4. Literature limited to one page; full text inaccessible; 5. Studies that lack clear diagnostic criteria, utilize self-designed efficacy assessment standards, or do not specify reference sources; 6. Research in which data extraction proved unfeasible or data were absent; duplicate publications and studies with incomplete data reporting were excluded.

### 2.3 Literature search

The overall databases include 4 English online data repositories consisting of PubMed, Web of Science, Cochrane Library, and Embase database, and 4 Chinese language databases constituting CNKI (China National Knowledge Infrastructure), CBM (Chinese Biomedicine), and the WanFang Database and Chinese Scientific Journals Database (VIP). Search for RCTs on acupuncture treatment for CAG, with a search period from 1 January 2000 to 1 August 2025. Chinese search terms included: “针灸疗法” (acupuncture therapy), “针灸治疗” (acupuncture treatment), “针刺治疗” (acupuncture treatment), “针刺疗法” (acupuncture therapy), “温针灸” (warm needle moxibustion), “电针” (electroacupuncture), “穴位埋线” (acupoint catgut embedding), “穴位注射” (acupoint injection), “耳针” (auricular acupuncture), “火针” (fire needling), “萎缩性胃炎” (atrophic gastritis), “慢性胃炎” (chronic gastritis). English search terms included “acupunture therapy” “acupuncture treatment” “electro-acupuncture” “acupoint injection” “Needle warming moxibustion” “Pharmacopuncture” “acupuncture and moxibustion” “Gastritis, Atrophic” “Gastrides, Atrophic” “Atrophic, Gastritis” “Atrophic, Gastritids.”

Using PubMed as an illustration:

1#(((((Gastritis, Atrophic [MeSH Terms]) OR (Atrophic Gastritides [Title/Abstract])) OR (Atrophic Gastritis [Title/Abstract])) OR (Gastritides, Atrophic [Title/Abstract])) OR (chronic atrophic gastritis [Title/Abstract])) OR (CAG [Title/Abstract])

2#((((((((((((((Acupuncture Therapy [MeSH Terms]) OR (Acupuncture Treatment [Title/Abstract])) OR (Acupuncture Treatments [Title/Abstract])) OR (Treatment, Acupuncture [Title/Abstract])) OR (Therapy, Acupuncture [Title/Abstract])) OR (Pharmacoacupuncture Treatment [Title/Abstract])) OR (Treatment, Pharmacoacupuncture [Title/Abstract])) OR (Pharmacoacupuncture Therapy [Title/Abstract])) OR (Therapy, Pharmacoacupuncture [Title/Abstract])) OR (Acupotomy [Title/Abstract])) OR (Acupotomies [Title/Abstract])) OR (electro-acupuncture [Title/Abstract])) OR (acupoint injection [Title/Abstract])) OR (Needle warming moxibustion [Title/Abstract])) OR (acupuncture and moxibustion [Title/Abstract])

3#(((random [Text Word] OR randomized [Text Word]) OR control [Text Word]) OR controlled [Text Word]) OR trial [Text Word]

1# AND 2# AND 3#

Retrieval utilizes a mesh database of words and free-text terms, with strategies modified based on the specific database.

### 2.4 Literature screening and data extraction

Two researchers conducted independent literature screenings, extracted data, and cross-verified findings; discrepancies were addressed through consultation with steering committee experts. The literature management was conducted using EndNote X9 software, and data extraction and analysis were performed with Excel 2016. The extracted content includes: - Fundamental study details (title, first author, publication year, journal, ethics review, trial registration); - Information on the study population (sample size, Hp infection status, gender ratio, age); - Treatment group characteristics (intervention method, treatment duration, acupoint prescription); - Control group characteristics (intervention method, treatment duration); - Primary and secondary outcome measures (measure name, primary outcome measurement time points, etc.); - Methodological characteristics (randomization method, allocation concealment, blinding, etc.).

### 2.5 Bias risk assessment

The Cochrane risk of bias assessment tool was utilized to assess the risk of bias in the studies included in the analysis. Evaluation items comprised random sequence generation, allocation concealment, blinding of patients and personnel, blinding of outcome assessors, incomplete outcome data, selective reporting of outcomes, and additional biases. Items were classified as having a “low risk of bias,” “uncertain risk of bias,” or “high risk of bias.”

### 2.6 Statistical analysis

All extracted data were compiled and analyzed using Excel 2016 software, while the outcome measures included in the study were standardized. In accordance with the Technical Specifications for Developing Core Outcome Measure Sets for Traditional Chinese Medicine Clinical Trials (10), outcome measures were classified into seven domains according to their functional attributes, illustrated through a tree diagram. A frequency analysis and descriptive analysis were performed for each category of outcome measure.

## 3 Results

### 3.1 Literature screening process

**FIGURE 1:**
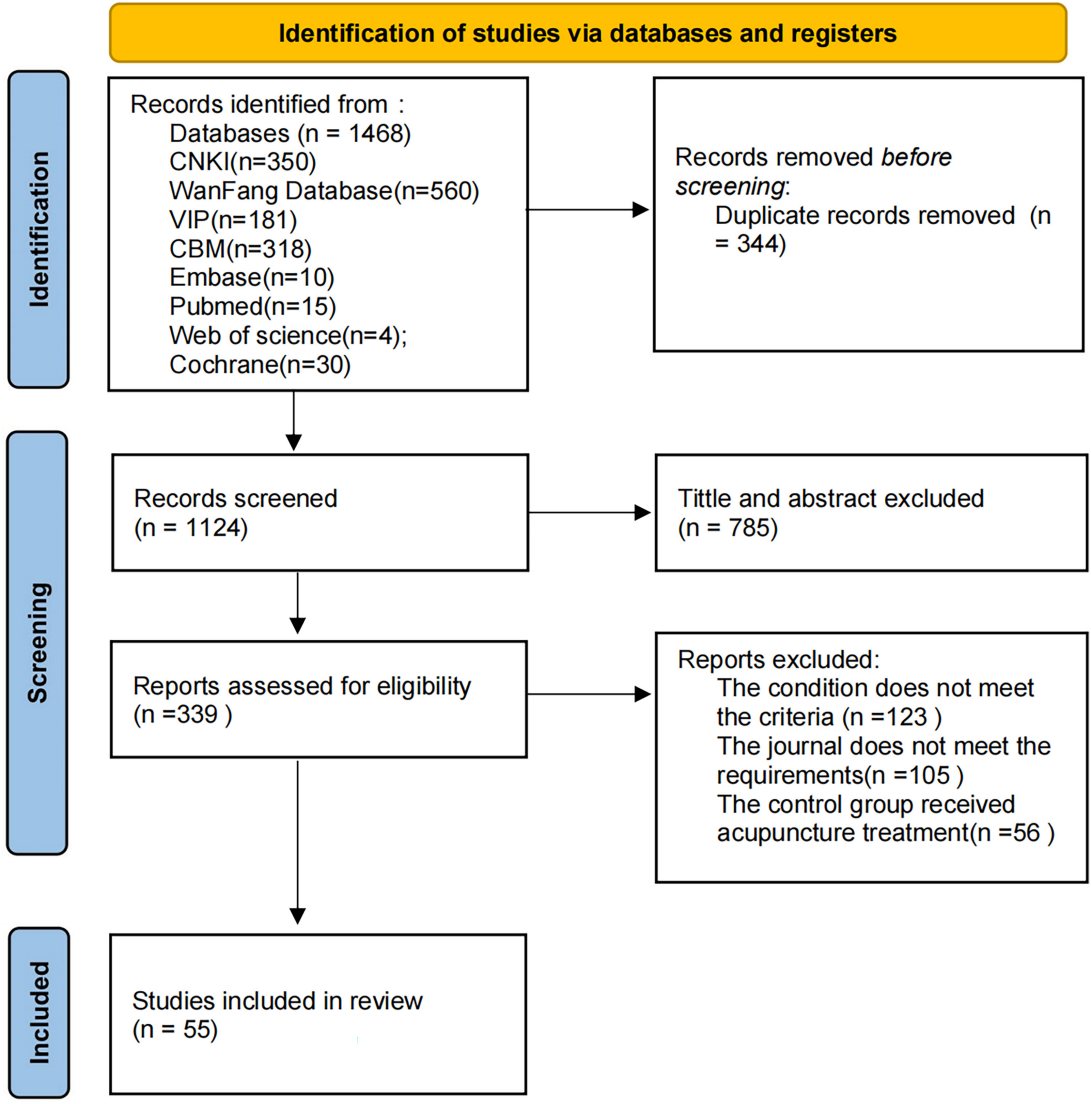
Literature screening flowchart.

A total of 1,468 articles were identified in the initial search. After a tiered screening process adhering to established inclusion and exclusion criteria, a total of 55 articles were retained: 55 in Chinese and none in English. Figure 1 illustrates the literature screening flowchart.

### 3.2 Basic characteristics of the included studies

Among the 55 Chinese-language publications analyzed, 14 (25.45%) were found in CSCD and Chinese Core Journals, 4 (7.27%) were listed in Chinese Core Journals, and 37 (67.27%) were published in Chinese Science and Technology Core Journals.

### 3.3 Sample size

A total of 5,311 patients participated, with 2,566 assigned to the intervention group and 2,745 to the control group. The sample sizes of individual studies varied from 51 to 202 participants, yielding an average of 97 across the studies analyzed.

### 3.4 HP infection status

Among the 55 included RCTs, 32 (58.19%) specified H. The status of H. pylori infection is included in the criteria for inclusion and exclusion. Of the 32 studies that specified H. pylori status, 18 (32.73%) included Hp-positive patients, 6 (10.91%) included Hp-negative patients, 8 (14.55%) included both Hp-positive and Hp-negative patients, and 23 (41.82%) did not specify the Hp infection status of the study subjects.

### 3.5 Interventions and control measures

The types of interventions encompassed acupuncture, moxibustion, electroacupuncture, warm moxibustion, acupoint plaster application, acupoint thread implantation, and combined Chinese herbal decoctions. The leading three interventions identified were acupuncture in conjunction with conventional Western medicine or Chinese herbal decoctions (21 studies, 38.19%), moxibustion (10 studies, 18.19%), and acupoint thread implantation (9 studies, 16.36%). Three randomized controlled trials (5.45%) reported two or more types of controls. Table 1 presents the types of controls for acupuncture treatments that were reported two or more times.

**TABLE 1:** Types of CAG controls with acupuncture treatment reported ≥ 2 times.

Comparison type	Frequency (proportion)/ occurrence (%)
Acupuncture + Chinese herbal decoction + WM vs. WM	5(9.10)
Acupuncture + Chinese herbal decoction vs. WM	3(5.45)
Acupuncture + WM vs. WM	3(5.45)
Acupuncture point thread implantation + Chinese herbal decoction + WM vs. WM	2(3.64)
Acupuncture point thread implantation + Chinese herbal decoction vs. Chinese herbal decoction	2(3.64)
Moxibustion + Chinese herbal decoction vs. WM	2(3.64)

WM, conventional western medicines.

### 3.6 Treatment course and follow-up arrangements

The studies reported treatment durations varying from 1 to 24 weeks. Six studies (10.91%) reported treatment durations of 2 weeks or less, seven studies (12.73%) had durations ranging from 2 to 4 weeks, 35 studies (63.64%) lasted between 4 and 12 weeks, and seven studies (12.73%) had durations of 12 weeks or more. Six studies (10.91%) provided follow-up data, with follow-up durations varying from 8 weeks to 1 year.

### 3.7 Bias risk assessment

The assessment of bias risk in the included studies was conducted utilizing the Cochrane risk of bias tool (11, 12). All 55 studies indicated the use of “randomization” in the generation of random sequences. Forty-six studies (78.00%, 83.64%) were classified as having a “low risk of bias” regarding random sequence generation. Of the studies analyzed, 46 (83.64%) utilized the “random number table method” for allocation, whereas 6 studies (10.91%) applied either the “Excel random table method” or the “SAS software random number generation method.” In six studies (10.91%), randomization was conducted using Excel randomization tables and SAS software-generated random numbers. Three studies (5.45%) were classified as having a “high risk of bias,” which included one study (1.82%) employing “lottery selection,” one study (1.82%) utilizing “odd/even appointment numbers,” and one study (1.82%) implementing “simple randomization by casting lots.” Allocation concealment: Two studies (3.64%) were assessed as having a “low risk of bias” for allocation concealment through the use of “sealed envelopes.” In the analysis of patient and personnel blinding, one study (1.82%) utilized a “double-blind” approach, categorized as “low risk of bias.” Another trial (1.82%) applied “single-blinding” but did not clarify whether investigators or outcome assessors were blinded. The remaining 53 trials (96.36%) were classified as having an “unclear risk of bias.” Outcome assessor blinding was not explicitly mentioned in 54 trials (98.18%), resulting in a classification of “high risk of bias.” Incomplete outcome data: Four studies (7.27%) explicitly documented patient dropouts. Of the studies examined, one (1.82%) exhibited dropout rates below 10% of the total sample size, categorizing it as having a “low risk of bias.” Conversely, three studies (5.45%) had dropout rates exceeding 10% of the total sample size, classifying them as having a high risk of bias. A total of 51 studies (92.73%) did not explicitly report patient attrition, resulting in an assessment of “risk of bias uncertain.” Selective reporting of outcomes: All 55 studies (100.00%) lacked access to prior study protocols, which precluded outcome comparisons and were assessed as having an “uncertain risk of bias.” Other biases: Sources of bias, including funding support and conflicts of interest, could not be identified, resulting in all being rated as “risk of bias: unclear.” The results were presented in Supplementary Figure 1.

### 3.8 Outcome indicator

#### 3.8.1 Indicator domain

**FIGURE 2:**
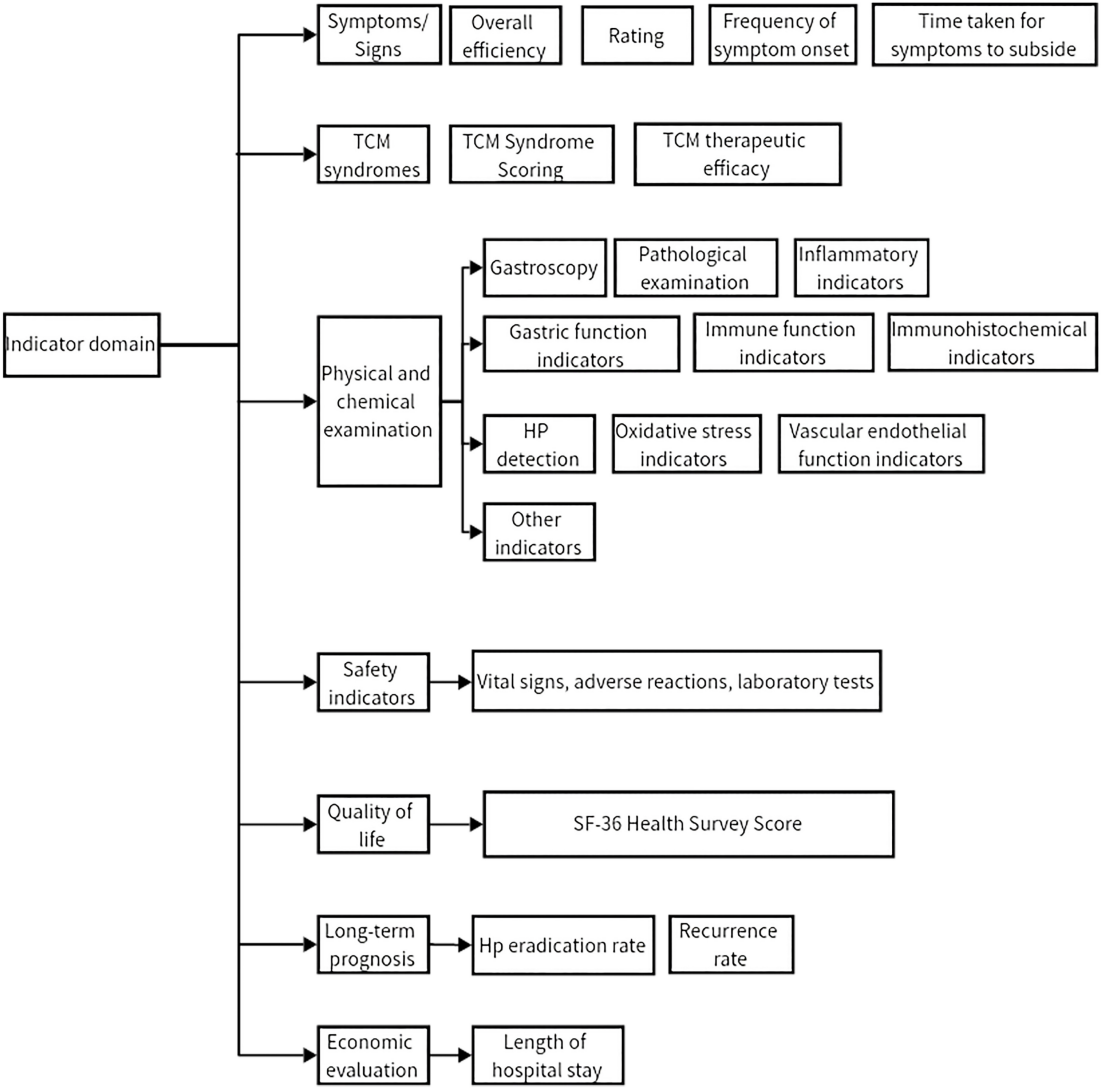
Indicator domain for acupuncture treatment of CAG.

A total of 57 outcome measures were reported across the 55 included RCTs, resulting in a combined frequency of 285 applications. Individual randomized controlled trials (RCTs) reported between one and fourteen measures, with an average of five measures per trial. Outcomes were classified based on functional attributes into seven domains, ordered by the frequency of their application: - Laboratory tests: 158 occurrences (55.44%) Symptoms and signs were observed in 51 instances, representing 17.89% of the total cases. Instances of traditional Chinese medicine syndromes: 44 (15.44%) Safety indicators: 23 occurrences (8.10%)— Long-term prognosis: 6 occurrences (2.11%)—Quality of life: 2 occurrences (0.70%)—Economic evaluation: 1 occurrence (0.35%) Laboratory test indicators were classified into 10 categories, and symptoms and signs were similarly categorized into 10 groups. Long-term prognosis was mentioned six times (2.11%), quality of life was referenced twice (0.70%), and economic evaluation appeared once (0.35%). Physicochemical examination indicators included 10 categories, while symptoms and signs encompassed 4 categories. Traditional Chinese medicine syndromes and long-term prognosis indicators each consisted of 2 categories. Additionally, safety indicators, quality of life, and economic evaluation indicators each comprised 1 category, as shown in Figure 2.

#### 3.8.2 Indicator application frequency

The application frequency of 57 outcome indicators was recorded, with the leading three identified as biochemical examination indicators, symptom and sign indicators, and traditional Chinese medicine syndrome indicators (detailed information was listed in Supplementary Table 1). The most frequently applied indicators included gastric function indicators, overall effective rate, and traditional Chinese medicine syndrome scores, occurring 48 times (16.84%), 41 times (14.39%), and 37 times (12.98%), respectively. Table 2 presents the frequency of outcome measures utilized across various domains of CAG in acupuncture treatment.

**TABLE 2:** Frequency of application of acupuncture treatment for outcome measures across CAG domains.

Indicator domain	Indicator name	Name of the next-level indicator	Frequency (proportion)/occurrence (%)	Indicator domain	Indicator name	Name of the next-level indicator	Frequency (proportion)/occurrence (%)
Physical and chemical examination	Pathological examination	Pathological efficacy	9 (3.16)	Physical and chemical examination	Immunohistochemical indicators	CEA, CDX2, Villin, and Ki67	1 (0.35)
		Efficacy of intestinal metaplasia treatment, pathological grading	1 (0.35)			GST-π, COX-1 and VR	1 (0.35)
		Therapeutic efficacy for glandular atrophy	3 (1.05)			Wnt3/β-catenin	1 (0.35)
		Glandular Atrophy Score	1 (0.35)				
		Intestinal metaplasia score	1 (0.35)		Vascular endothelial function indicators	Epidermal Growth Factor (EGF)	1 (0.35)
		Atypical Hyperplasia Score	1 (0.35)			Vascular endothelial growth factor (VEGF)	2 (0.70)
		Chronic Inflammation Score	1 (0.35)			Human basic fibroblast growth factor (bFGF)	2 (0.70)
		Activity score	1 (0.35)				
		Pathological Grading of Intestinal Metaplasia	1 (0.35)		HP detection	HP eradication rate	15 (5.26)
		Pathological Grading of Atrophy	1 (0.35)			HP positivity rate	1 (0.35)
		Pathological Grading of Dysplasia	1 (0.35)		Gastroscopy	Gastroscopy efficacy	10 (3.51)
		Gastroscopy Grading, OLGA/OLGIM Scoring and Reversal Rate	1 (0.35)			Gastric mucosal scoring during gastroscopy	6 (2.11)
						Gastroscopic imaging changes	2 (0.70)
		Pathological grading	15 (5.26)		Oxidative stress indicators	Maldodicarbox- aldehyde (MDA)	2 (0.70)
						Superoxide dismutase (SOD)	2 (0.70)
	Gastric function indicators	Pepsinogen I (PG I)	9 (3.16)			Glutathione peroxidase (GSH-Px)	1 (0.35)
		Pepsinogen II (PG )	9 (3.16)				
		PGR (PG I PG II)	7 (2.46)		Other indicators	Serum TGF-α, CXCL10	1 (0.35)
		Gastrin (GAS)	10 (3.51)				
		Motilin (MLT)	4 (1.40)	TCM syndromes	TCM Syndrome Scoring	Syndrome Scoring System	14 (4.91)
		Somatostatin (SS)	8 (2.81)			Symptom Scoring System	23 (8.10)
		Vasoactive intestinal peptide (VIP)	1 (0.35)		Therapeutic Efficacy of TCM in Treating Diseases and Syndromes	Therapeutic Efficacy of TCM Syndromes	5 (1.75)
						Therapeutic Effects of TCM Symptoms	2 (0.70)
	Inflammatory indicators	Tumor Necrosis Factor-α (TNF-α)	7 (2.46)				
		Interleukin-6 (IL-6)	4 (1.40)	Symptoms and signs	Overall efficiency	Overall efficiency	41 (14.39)
		Interleukin-11 (IL-11)	2 (0.70)		Rating	VAS	3 (1.10)
		Interleukin-8 (IL-8)	5 (1.75)			Clinical Symptom Score	1 (0.35)
		Interleukin-17 (IL-17)	2 (0.70)			Frequency of stomach pain episodes	1 (0.35)
		Interleukin-10 (IL-10)	2 (0.70)			PRO Scale	2 (0.70)
		Neutrophil/lymphocyte ratio (NLR)	1 (0.35)			HAMA Scale	1 (0.35)
		Interleukin-1β (IL-1β)	1 (0.35)			Time taken for abdominal pain and bloating to subside	2 (0.70)
		Security incident	23 (8.10)	HP eradication rate, recurrence rate	
	Immune function indicators	CD4 + , CD8 + , CD4 + /CD8 +	1 (0.35)			Long-term prognosis	6 (2.11)
				Quality of life		SF-36 Health Survey Score	2 (0.70)
				Economic evaluation		Length of hospital stay	1 (0.35)

### 3.9 Overall efficiency

All 41 RCTs documented measurement time points, which varied from 2 weeks to 12 months. The most commonly reported time point was 3 months post-treatment, noted in 18 studies (33.33%). Table 3 presents the measurement time points for the overall response rate across the included studies.

**TABLE 3:** Time points for measuring the overall response rate to acupuncture treatment for CAG.

Measurement point	Frequency (proportion)/ occurrence (%)	Measurement point	Frequency (proportion)/ occurrence (%)
2 weeks	1 (1.90)	10 weeks	1 (1.90)
4 weeks	9 (16.67)	16 weeks	3 (5.56)
8 weeks	13 (24.07)	24 weeks	7 (12.96)
12 weeks	18 (33.33)	One year after treatment	2 (3.70)

### 3.10 Traditional Chinese Medicine (TCM) symptom scoring system

All 23 RCTs documented measurement timepoints, with each conducting assessments both prior to and at the conclusion of treatment. Two randomized controlled trials conducted follow-up assessments after treatment. The predominant measurement timepoints reported were pre-treatment and 2 months post-treatment, as indicated in 9 studies, accounting for 32.14% of the total. Table 4 presents the measurement timepoints for TCM symptom scores across the included studies.

**TABLE 4:** Timepoints for measuring CAG TCM symptom scores following acupuncture treatment.

Measurement point	Frequency (proportion)/Occurrence (%)	Measurement point	Frequency (proportion)/Occurrence (%)
BT, 2 weeks	1 (3.57)	BT, 12 weeks	7 (25.00)
BT, 4 weeks	6 (21.43)	BT, 24 weeks	3 (10.71)
BT, 6 weeks	1 (3.57)	BT, 1 year after treatment	1(3.57)
BT, 8 weeks	9 (32.14)		

BT, Before treatment.

## 4 Discussion

There has been some progress in clinical research on acupuncture treatment for chronic atrophic gastritis, but large-scale clinical trials remain scarce. The methodological quality of contemporary studies is frequently insufficient, marked by a deficiency of high-quality publications with considerable international influence. The efficacy demonstrated in certain research does not sufficiently substantiate the therapy’s general applicability or its merit for widespread adoption. Furthermore, a high frequency of clinical use does not inherently indicate a robust level of evidence. Clinical research is important because it helps doctors do their jobs better and improves patient health. So, doing high-quality clinical research is very important for making sure that medicines work and are safe. It is very important to have a strict study design to make sure that science is reliable and trustworthy. The appropriate utilization of outcome measures in study design is essential, since it directly affects the validity and therapeutic significance of research findings. In clinical trials, including traditional Chinese medicine, it is typical to see differences in the choice of outcome measures and reporting that is not standardized. This diversity makes it harder to understand and compare the results of different studies, and it also limits the scope of future secondary research, such as systematic reviews and meta-analyses. This kind of variety lowers the overall quality and level of evidence (13). The study provides a comprehensive overview of intervention designs, outcome measure frequencies, and measurement timepoints, whose results indicate that existing studies exhibit significant design deficiencies, especially with the formulation of outcome measures.

It is imperative that the efficacy and scientific validity of acupuncture therapy be evaluated and interpreted using internationally accepted research methodologies and evaluation standards. Consequently, it is essential to standardize clinical research designs for CAG acupuncture treatment. Five important parts make up the PICOS framework, which is suggested as a main strategy for systematic reviews: participants, intervention, comparison, result, and research design. This is a necessary basis for performing high-quality clinical research (14, 15). Following the PICOS principles from the beginning makes trials more rigorous and their results more believable.

This paper will follow the PICOS principle, focusing on the design challenges associated with acupuncture clinical trials for chronic atrophic gastritis. This study will concentrate on the standardized establishment of outcome measures, which will entail a detailed investigation and analysis, as described below.

### 4.1 Research overall design

The design of a study must start with clearly defined research goals, which set the basic structure of the study and the amount of proof that is needed. Multicenter, double-blind randomized controlled trials employing superiority or non-inferiority hypotheses, along with prospective real-world research, should be prioritized in efficacy evaluations. To keep the quality of the trial high, it is important to use blinding. If blinding is not possible, the procedure must offer a detailed explanation and outline the alternative steps taken to deal with this issue. The research protocol must explicitly and fully explain all of the main design ideas, using the PICOS framework.

### 4.2 Research registration gaps and insufficient awareness of ethical review

Research registration and research ethics constitute the twin pillars safeguarding scientific quality. The former endeavors to promote transparency throughout the research process by publicly disclosing protocols and progress, thereby preventing publication bias and selective reporting that upholds the objectivity of the scientific record. The latter serves as the ethical compass guiding research practice, establishing essential value boundaries for research activities. The significance of both has been legally enshrined: pursuant to the 2019 Basic Medical and Health Care and Health Promotion Law of the People’s Republic of China, all biomedical research involving human subjects must undergo ethical review. This signifies that the transparency advocated by research registration and the fairness upheld by ethical review collectively form the robust foundation of responsible research in China (16–18). Analysis of the 55 randomized controlled trials (RCTs) included in this study revealed significant deficiencies in reporting research registration and ethical review information. Specifically, none of the studies (100.00%) provided research registration details, while over half (65.45%, 36 studies) failed to report ethical review information. This situation reflects that, within the manuscript review and publication processes of certain academic journals in China, formal review requirements for research registration and ethical review may be relatively lax, leading to insufficient emphasis on these aspects by researchers. To fundamentally enhance the scientific value and credibility of research, it is strongly recommended that researchers complete study registration on internationally recognized platforms such as ClinicalTrials.gov and obtain approval from an ethics review committee prior to formally commencing their research. During implementation, timely updates on research progress should be maintained on the registration platform. Upon publication, studies must explicitly state their registration number and ethics approval number within the manuscript. Establishing this comprehensive, transparent management system will effectively curb selective reporting bias, ensuring every stage of research—from design to publication—stands up to scrutiny.

### 4.3 Identify the research subjects (P)

The initial phase of designing a clinical trial involves delineating the research subjects. The primary concept of clinical trials is that the research subjects consist of individuals, particularly patient populations, as opposed to experimental studies that utilize *in vitro* materials such as cells or tissues (19). This fundamental aspect necessitates that researchers meticulously consider the potential impact of social and psychological factors on the trial process and outcomes while emphasizing the safety and rights of participants. The inclusion criteria must align with the latest, authoritative national or global guidelines for disease diagnosis, treatment recommendations, standards, and expert consensus. This means that the criteria for choosing subjects need to be set up very carefully. Exclusion criteria are meant to weed out groups that already fit the inclusion criteria, as well as those who offer higher risks, add confusing variables, or don’t meet particular trial requirements. It is important to stress that exclusion criteria are not only the opposite of inclusion criteria; they are a required enhancement and addition to them (20).

### 4.4 Intervention (I) setup

The scientific and systematic design of interventions and control measures is essential for ensuring internal validity, outcome dependability, and clinical generalizability in research (21). Acupuncture intervention is a complex multidimensional system that incorporates important components such as point formulations, needling techniques, treatment frequency, and course duration. The examination of the 55 RCTs included in this study revealed that existing intervention protocols had ambiguous and non-standardized descriptions, as well as a lack of reproducible operational details, limiting generalizability and validation of conclusions. Acupoint selection is typically based on Traditional Chinese Medicine syndrome differentiation (e.g., spleen-stomach insufficiency, liver-stomach heat stagnation), with primary points such as Zusanli (ST36), Zhongwan (CV12), Weishu (BL21), and Pishu (BL20). However, there is a substantial discrepancy in studies on point combination concepts and point modification criteria. Future studies should strictly adhere to the Standard Reporting of Clinical Trials of Acupuncture and Moxibustion (STRICTA), which requires exhaustive reports of acupoint lists and selection rationale, including standard international nomenclature (e.g., ST36) and localization references for all used points, as well as explicit statements about whether selection is based on classical theory, expert consensus, or contemporary research. When employing syndrome differentiation, offer specific diagnostic criteria (for example, the Consensus on Diagnosis and Treatment of Chronic Atrophic Gastritis in Traditional Chinese Medicine). In agreement with international high-quality acupuncture clinical research guidelines, a “semi-fixed” acupoint selection paradigm is recommended for trials. When protocols include individualized point combinations, clear pattern differentiation modification criteria must be established (for example, stating that particular points must be added if concurrent symptoms such as “epigastric burning pain, dry mouth, and bitter taste” arise). Therefore, it keeps clinical practice flexible while ensuring protocol reproducibility and scientific rigor.

In acupuncture, the existence or absence of deqi feeling, as well as the use of tonifying or dispersion procedures, are critical elements impacting therapeutic success. However, present research primarily addresses procedures such as “balanced tonification and dispersion” or “needle retention for 30 min,” which lack objective quantification. Key techniques (such as twisting, raising, and thrusting) should be stated in terms of frequency, amplitude, and duration. Standardized method training and assessment should be considered to ensure uniformity among practitioners. Given the variety of acupuncture therapies, appropriate procedures should be chosen based on applicable criteria and clinical expertise. Simultaneously, attempts may be made to quantify stimulation variables. For example, when practitioners use electroacupuncture, they must precisely describe the waveform (continuous/alternating/intermittent), frequency, current intensity, and adjustment principles. Needle specifications (diameter, length) should be indicated, as should the necessity for adjunct therapies such as moxibustion (e.g., heated needle moxibustion) or infrared irradiation, as well as their precise operational parameters. In terms of treatment frequency and length, CAG is a chronic pathological change that requires extensive cumulative therapy to reverse. An overly brief treatment may prevent adequate evaluation of acupuncture’s long-term effects.

Only a small percentage of studies (12.73%) had treatment durations longer than 12 weeks, whereas the bulk of clinical trials (63.64%) specified treatment durations between 4 and 12 weeks. Additionally, a follow-up observation period following therapy termination was absent from the majority of investigations. Such a pattern has led to a dearth of evaluation of the long-term effectiveness and durability of acupuncture therapy for CAG, which raises questions about whether acupuncture can reverse stomach mucosal disease in such a short amount of time. The physiological window for improvement in gastric mucosal pathology and previous feasibility studies should be considered when designing the length of treatment. Establishing a core therapy term of at least 12 weeks is advised, and it is strongly advised to extend it to roughly 24 weeks to cover the probable pathological improvement time window (22). To differentiate between short-term impacts and long-term efficacy, a follow-up period of at least 6 months after therapy must be established concurrently. A more scientifically sound foundation for assessing whether acupuncture may treat the underlying cause—that is, reversing atrophic lesions in the stomach mucosa—is provided by such a design. The biological time window for pathological healing and the patient’s bearable burden must be considered while determining the “therapeutic dose” of acupuncture intervention, which is the sum of the treatment frequency and duration. It is challenging to evaluate acupuncture’s long-term effects on gastric mucosal pathology because the majority of current research had relatively short treatment durations (≤ 12 weeks) and no follow-up. Theoretically, idealized high-frequency treatments (such as three or more sessions per week) could increase efficacy, but in long-term reality, patient compliance is a problem. When selecting how often to treat someone, you need to carefully weigh the theoretical efficacy criteria and the clinical feasibility. Some theoretical or rigorous therapy protocols prefer high-frequency stimulation three to five times a week. However, these high-frequency regimens are often not possible for long-term management of diseases like CAG because they take up too much of the patient’s time and are not followed through on. This, however, would increase the number of people who quit, which would make the study less useful to subsequent studies. So, frequencies that are clinically useful for general use (such as twice to three times a week) should be given priority, as long as they still work well. The study protocol must explicitly articulate the rationale for this frequency determination (e.g., grounded in prior feasibility studies or patient burden evaluations). To find the “minimum necessary frequency” for maintaining efficacy and finding the best balance between efficacy and feasibility, future studies may divide participants into groups with varying frequencies. A “stepwise” design technique might be employed instead of just one high-frequency model. This would look at flexible timetables like “an initial intensive phase (e.g., three times a week for the first four weeks) followed by a maintenance phase (one to two times a week).” This technique would be more in line with clinical practice.

### 4.5 Configuration of countermeasure (C)

To tell the difference between the advantages of acupuncture and the effects of a placebo or doctor-patient contact, you need to use good controls. The controls for the CAG acupuncture study must find a compromise between scientific integrity and clinical ethics. Common controls include blank or waiting list controls, sham acupuncture controls (like shallow needling at non-meridian points or puncturing non-acupoint locations), positive medication controls such as folic acid or traditional Chinese patent medicines and simple preparations, and controls that compare different acupuncture procedures. To prove that acupuncture works, you could utilize positive pharmacological controls or loading designs, like “standard drug therapy + acupuncture” versus “standard drug therapy + sham acupuncture/blanking.” This method is helpful for therapy, even though it’s hard to tell the difference between non-specific effects. To find out what effects acupuncture has on the body, use phony acupuncture controls. Fake acupuncture should look and feel like real acupuncture, but the needling or stimulation should not work. To find out if it works, blind participants must be tested. Placebo controls can measure all of acupuncture’s benefits, but expectations may affect the results. Whenever you can, use them as extra controls. Although sham acupuncture is the gold standard in terms of methodology, there are several obstacles to clinical study, such as the absence of widely accepted, completely non-biologically active sham sites and the possible physiological consequences of superficial needling. Priority should be given to choosing superficial sites for sham needling that are far from well-known meridians and acupoints and where initial research shows no efficacy for the target condition (23). Alternatively, placebo needles, like Streitberger needles, may be taken into consideration. These have blunt points that retract when they come into contact with the skin, giving the impression that they are being inserted without actually penetrating. Both the practitioner and the subject can double-blind more successfully because of this design.

### 4.6 Set outcome indicators (O)

Clinical research is guided and valued by outcome measurements. Following a systematic analysis of 55 RCTs, current acupuncture studies for chronic gastritis have significant outcome measure selection issues, compromising reliability, comparability, and clinical translation value. Three dimensions show the main issues: First, indicator selection is “excessive and disparate,” comprising dozens of symptom scores, gastroscopy, pathology, serology, and quality-of-life ratings. These indicators’ applicability rates vary widely, with little consensus. Second, indicator implementation is “disorganized and ambiguous”; even identically called metrics (such as “clinical total effective rate”) have different assessment and calculation criteria. Key indications like histological grading are measured inconsistently, either at treatment completion or months later, making it impossible to discern immediate impacts from long-term efficacy. Finally, the indicator framework confuses primary and secondary outcomes. Most studies confuse accessible but clinically limited subjective symptom scores with objective pathological indications of disease essence, leading to ambiguous findings.

To improve the scientific rigor and evidence strength of future research, the optimization of outcome measures should focus on clinical value, prioritize objective pathology, and ensure standardized reporting.

### 4.7 Promoting the establishment of a core set of outcome measures for acupuncture treatment of CAG

The Core Outcome Set (COS) refers to a fundamental, essential collection of outcome measures that must be assessed and reported in all clinical trials pertaining to specific health domains or diseases. The aim is to address the fragmentation of evidence resulting from inconsistencies in outcome selection, definitions, and measurement methods across studies, while not restricting researchers from exploring additional measures. This facilitates effective comparison, synthesis, and meta-analysis of results from various studies, thereby augmenting the overall value of the evidence base (24). The evaluation of CAG as a precancerous lesion necessitates three dimensions: symptoms, function, and pathology. Future research should prioritize the implementation of the proposed structured indicator system and foster consensus throughout the discipline. Primary outcome measurements must delineate critical variables that directly and objectively reflect the fundamental aspects of CAG. Gastric mucosal histological scoring, particularly the enhanced OLGA/OLGIM staging system, should be regarded as the primary indicator, as it effectively assesses atrophy, reverses intestinal metaplasia, and mitigates cancer risk (25, 26). When the primary objective of a study is symptom improvement, it is crucial to employ meticulously vetted patient-reported symptom assessment measures. The assessment of symptomatic efficacy may utilize measures such as the 7-point Global Outcome Symptom Scale (GOSS), the Gastrointestinal Symptom Rating Scale (GSRS), or the Functional Dyspepsia Symptom Diary (FDSD) (27). For a complete evaluation, secondary outcomes must be thoroughly combined. This includes quantifiable biological markers like the serum pepsinogen I/II ratio (PGR) and gastrin-17 levels, which are blood tests that show how well the stomach lining is working. It also includes quality of life assessments and safety indicators, using universal scales like the SF-36 or disease-specific tools like the Gastrointestinal Quality of Life Index (GIQLI), as well as systematic records of adverse events and responses.

### 4.8 Strictly implement standardized definitions and measurement protocols for indicators

All outcome measures must adhere to strict, standardized definitions and measurement procedures to guarantee the validity, repeatability, and comparability of study findings across investigations. Ambiguous indicator definitions and opaque measurement methods and methodologies are a prevalent problem in contemporary research, making it difficult to integrate and analyze “heterogeneous outcomes under the same name.” First and foremost, every outcome measure needs to have a precise operational description that includes the source and justification for measurement. For example, when using “pathological histological response rate,” the response threshold (e.g., defined as a reduction of at least one grade in the degree of atrophy or intestinal metaplasia from baseline) must be precisely defined along with the underlying pathological scoring system (e.g., the New Sydney system). The majority of studies only describe “pathological improvement” without offering a precise definition, making the findings inappropriate for scientific comparison and assessment. Second, to guarantee data quality, standardization of measurement instruments, procedures, and operators is essential. This includes objective indicators: for example, information on the biopsy site, number of biopsy specimens, gastroscope model, and histological staining techniques (e.g., specific stains, hematoxylin and eosin staining) should be provided in the context of gastroscopy and histopathological examination. Laboratory indicators: identify the kit manufacturer, instrument model, and assay technique (such as ELISA) for serum pepsinogen; Subjective indicators: standardize survey procedures (such as in-person interviews conducted by qualified investigators in quiet environments) for patient-reported outcome (PRO) scales, make sure that instructions are uniform, and apply data quality control. Lastly, every important outcome measure needs to be pre-specified with precise assessment timepoints and a justification based on science. The expected extent of the intervention effect and the disease’s natural history should be considered when choosing a timepoint. At the very least, a thorough evaluation timeline should comprise the following: baseline, important treatment milestones, treatment completion, and primary follow-up dates after treatment. To prevent selective reporting bias and allow for the creation of a comprehensive time-to-effect curve, all time points must be pre-registered in the trial protocol and completely provided in the study report.

### 4.9 Design research type (S)

A scientifically sound study design makes it possible to gather reliable evidence. Most of the current clinical evidence on acupuncture for CAG is based on tiny, single-center randomized controlled trials (RCTs). These show major flaws in design rigor, evidence quality, and the justification of research phases, even when they show that the treatment works in the first place. Most studies do not create a complete evidence translation chain because they are still in the “exploratory” stage and do not have “confirmatory” research with a big sample size to fill the gap. The progression of clinical evidence should follow a logical sequence, from preliminary research to definitive validation. The early exploratory phase aims to enhance intervention protocols and assess feasibility through high-quality single-arm before-and-after studies or small-sample randomized controlled trials (RCTs). To provide precise parameter estimates for future research, the emphasis is on meticulously adhering to SPIRIT-A and STRICTA standards for protocol design, detailing intervention specifics, and employing multiple outcome measures for comprehensive analysis (28).

The primary objectives of the midterm validation phase are to evaluate the effectiveness of the therapy and establish a foundation for estimating the sample size. It is advisable to conduct thorough, multi-center RCTs with adequate sample sizes. At this point, it’s very important to choose the right control measures. The goals of the study should help you choose between sham acupuncture controls and positive medication controls. Pre-registration of trial methods is mandatory, and techniques such as centralized randomization and allocation concealment must mitigate bias. The late-stage confirmation and effectiveness evaluation phase aims to generate robust evidence and assess practical outcomes. It is important to support large, multicenter efficacy RCTs or cluster-randomized controlled trials. Longitudinal studies or cohort studies utilizing registry data may be established to evaluate the long-term safety and efficacy of acupuncture treatment subsequent to the validation of RCTs.

Additionally, it is essential to fortify fundamental methodological elements during the research design phase. To get rid of selection bias, randomization should use computer-generated random sequences, and to keep the allocation secret, sealed opaque envelopes or an independent, unpredictable central randomization process should be used. During the blinding method, participants, outcome assessors, and data analysts should be blindfolded wherever feasible. To evaluate the efficacy of blinding in sham acupuncture controls, post-trial assessments of participants’ capacity to ascertain their allocation group are necessary. Statistical analysts are required to conduct primary analyses without prior knowledge of their allocated groups. When designing a hypothesis-testing study, calculations for sample size should consider the main outcome measure, the predetermined effect size, the α error, and the power level. It is important to keep accurate records of how the computation method works.

A high-quality study design needs strict rules for managing data. Electronic data collecting devices that obey the rules for handling clinical trial data will become a trend in the future. EDC systems use advanced logic checks and mandatory field rules in electronic case report forms (eCRFs) to drastically reduce data omissions, outliers, and logical errors at the source. The system lets users enter data in real time online. Their audit trail feature keeps track of all modifications to the data, making sure that the data is reliable and comprehensive while also following all rules for data traceability and Good Clinical Practice (GCP). After a thorough analysis of the CAG studies for acupuncture treatments that are currently enrolled, it was found that there is a pervasive lack of uniformity in how data is handled. Most of the studies used general-purpose software (such as Microsoft Excel) to process the data, and they didn’t use specialized clinical trial data management systems. Some research didn’t even include certain data management practices in their results, which could lead to biases, mistakes, and missing data. This technique is completely contrary to the goal of producing reliable clinical evidence.

## 5 Conclusion and outlook

This study conducted a systematic analysis of RCTs examining acupuncture for CAG from 2000 to 2025, highlighting notable methodological limitations in the current evidence. Significant issues identified were the prevalent lack of prospective trial registration and ethics review documentation; vague and inadequately standardized descriptions of interventions (including acupoints, techniques, and treatment duration); an abundance of outcome measures characterized by inconsistent terminology and unclear prioritization, coupled with a deficiency in patient-centered and long-term prognostic evaluations; and basic data management practices, often dependent on non-specialized tools. The identified deficiencies significantly compromise the internal validity of individual studies and hinder the comparison, synthesis, and application of findings across research. The primary issue is the lack of a standardized framework for designing clinical research protocols, particularly the absence of unified and universally recognized standards for outcome measures. The main objective of facilitating a paradigm shift in acupuncture clinical research for CAG is to establish and advocate for a “core outcome set” (COS) and a “standard clinical trial design protocol” within this domain.

This systematic review of acupuncture for chronic atrophic gastritis (CAG) over the past 25 years reveals that despite the accumulation of literature, significant deficiencies in methodological rigor and standardization of outcome measures persist, resulting in fragmented data that hinders the generation of high-quality evidence. Compared to pharmacological and surgical RCTs in gastroenterology, acupuncture research lags in key bias control measures; while drug trials often achieve double-blinding and surgical trials frequently employ independent review committees, only 1.8% of the included acupuncture trials implemented blinding (particularly assessor blinding), and allocation concealment was reported in merely 3.6% of studies. Furthermore, the current literature exhibits a heavy reliance on laboratory and imaging examinations (accounting for 55.4% of outcomes) while neglecting patient-reported outcomes, health economic evaluations, and long-term prognosis. This misalignment between surrogate markers and clinical benefits limits the translational value of current findings. Consequently, there is an urgent need to establish a Core Outcome Set (COS) for acupuncture in CAG and to conduct multicenter, large-sample RCTs that strictly adhere to CONSORT guidelines and include long-term follow-up, thereby advancing the standardized development of acupuncture practice and producing internationally credible evidence.

It is important to remember the study’s limitations when looking at the results. To maintain a focus on published data utilizing standardized methodologies, the literature search was confined to essential Chinese and English-language journals, excluding grey literature and clinical trial registry protocols. This feature may lead to publication bias and undermine the thoroughness of the results. Relevant studies from non-English speaking countries (e.g., Japan or Korea) might have been excluded due to language barriers and database constraints. The tiered screening process yielded only Chinese-language articles, with no English studies meeting the inclusion criteria. This linguistic homogeneity restricts international scientific validation, particularly as publication standards in some domestic journals may be less rigorous. The absence of studies in internationally recognized databases, such as the Web of Science Core Collection, highlights a critical gap in global academic recognition. To enhance credibility and generalizability, future research should prioritize publishing high-quality trials in international peer-reviewed journals to facilitate broader validation of acupuncture’s efficacy for chronic atrophic gastritis. Additionally, the current evidence is derived primarily from Chinese populations, and the outcome measures rely heavily on surrogate endpoints, such as laboratory examinations, with a notable lack of patient-reported outcomes and long-term prognosis assessment. Consequently, these factors limit the generalizability of the findings to other populations and clinical settings. Future multicenter, large-sample international collaborative studies are needed to validate the results of this review. Furthermore, given acupuncture’s status as a significant element of Chinese cultural heritage and the fact that all the studies analyzed in this review were conducted in China, the generation of more robust, confirmatory evidence would greatly benefit from the strategic involvement of Chinese governmental institutions responsible for science and technology. Such high-level support is crucial for establishing structured, large-scale research programs and a comprehensive framework capable of sustaining high-quality clinical investigations in this field. Second, while our risk of bias assessment adhered to Cochrane RoB 2.0 standards, the predominance of “high” or “some concerns” judgments in allocation concealment and blinding domains reflects well-documented methodological constraints in acupuncture research. Unlike pharmacological trials, acupuncture inherently limits practitioner and participant blinding due to the tactile and procedural nature of needle insertion. Even when sham acupuncture is employed, non-specific physiological effects (e.g., diffuse noxious inhibitory controls, localized microtrauma) may dilute true treatment contrasts, while inadequate allocation concealment in small, single-center trials frequently introduces selection bias. Meta-epidemiological evidence consistently demonstrates that trials with unblinded participants or inadequately concealed allocation overestimate treatment effects by 15–30%, particularly for subjective endpoints such as pain intensity, gastrointestinal symptom scores, and TCM syndrome evaluations. In the present review, this bias pattern likely contributes to the observed heterogeneity in symptom-related outcomes, whereas objective measures (e.g., histopathological grading, H. pylori eradication rates, inflammatory biomarkers) remain comparatively robust. To mitigate these limitations, future acupuncture trials should prioritize centralized randomization with opaque sealed envelopes or web-based systems, employ validated non-penetrating sham needles with rigorous credibility testing, and mandate independent, blinded outcome assessment for all primary endpoints. Transparent reporting of concealment and blinding procedures according to STRICTA and CONSORT guidelines is essential to enhance internal validity and improve the reliability of meta-analytic estimates in acupuncture research. Third, the study focused on qualitative summary and theoretical examination of current challenges, neglecting comprehensive quantitative analysis of usage frequency and measurement instrument consistency for each outcome indicator. The study did not perform comprehensive comparison analyses between various intervention subtypes (e.g., electroacupuncture versus manual acupuncture) or patient subgroups (e.g., individuals with and without intestinal metaplasia).

Future clinical research on acupuncture treatment for CAG should concentrate on the coordinated progress of the following three levels, as determined by the status of the field today and the systematic analysis carried out in this study: First, in order to provide a consistent, comparable methodological framework for all recently started investigations, a core set of outcome measures (COS) and standardized clinical research protocol reporting criteria should be established as soon as possible. Future trials should prioritize validated patient-reported outcome measures (PROMs), such as the Gastrointestinal Quality of Life Index (GIQLI) or the SF-36, to capture the holistic impact of acupuncture on patient wellbeing. Furthermore, given the nature of CAG as a precancerous lesion, long-term follow-up is essential; thus, we recommend including recurrence rates and histological progression-specifically the reversal of atrophy and intestinal metaplasia—as key prognostic indicators. To inform healthcare policy and clinical guidelines, future studies should also incorporate cost-effectiveness analyses to evaluate the economic value of acupuncture compared to standard pharmacological treatments. Incorporating these domains will ensure that future clinical trials generate evidence that is not only methodologically rigorous but also meaningful to patients and healthcare stakeholders. Second, based on the previously described COS, multicenter, large-sample, high-quality randomized controlled studies should receive priority funding. In order to produce high-quality and widely applicable evidence, the future multicenter randomized controlled trials on acupuncture and moxibustion treatment of chronic atrophic gastritis must comply with strict methods and ethical standards. First, in order to ensure scientific integrity, the trial must be prospectively registered on a recognized platform, such as the World Health Organization International Clinical Trial Registration Platform (WHO ICTRP) or its main registration centers (such as ClinicalTrials.gov, ISRCTN), and specific suggestions on using the International Traditional Medicine Clinical Trial Registration Center (ITMCTR) to improve the credibility of acupuncture and moxibustion research are proposed. Secondly, in order to ensure statistical validity, sample size calculations should be based on primary outcomes (such as symptom improvement or histological reversal), with a significance level of α = 0.05, efficacy of 80–90%, and an expected dropout rate of 10–15%, requiring large-scale collaboration. Thirdly, given the chronic nature and malignant potential of CAG, it is crucial to follow-up for at least 12 months after treatment. Endoscopic and pathological reassessment should be conducted every 6 and 12 months to evaluate long-term efficacy and histological progression. Finally, to ensure transparency, research must strictly adhere to the CONSORT statement and STRICTA checklist, detailing intervention plans and adverse event monitoring, while complying with the Helsinki Declaration and obtaining independent IRB/EC approval. By integrating these standards - robust registration, sufficient power, extended follow-up, and standardized reporting - research in this field can transition from fragmented studies to patient-centered high-level evidence.

## Data Availability

The original contributions presented in this study are included in the article/Supplementary material, further inquiries can be directed to the corresponding author.
