# Supplementary figures and images for: A methodological review of acupuncture for chronic atrophic gastritis: toward a core outcome set
Source: Front Med (Lausanne). 2026 Jun 1;13:1818918. doi: 10.3389/fmed.2026.1818918 (PMC13265282; doi:10.3389/fmed.2026.1818918)

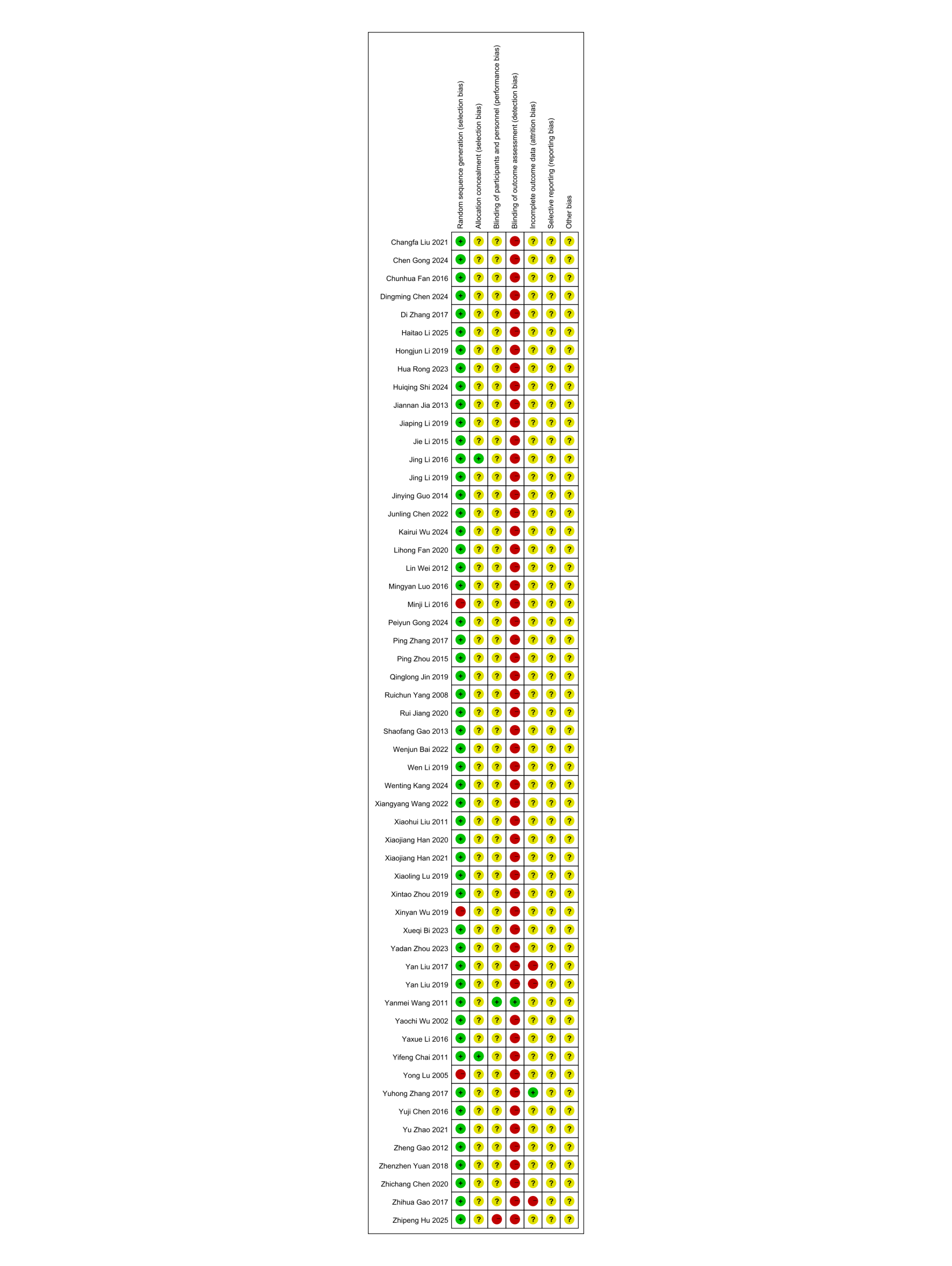

Supplement: Supplementary file 1 [file Image_1.tif]
