# Supplementary material for: A methodological review of acupuncture for chronic atrophic gastritis: toward a core outcome set
Source: Front Med (Lausanne). 2026 Jun 1;13:1818918. doi: 10.3389/fmed.2026.1818918 (PMC13265282; doi:10.3389/fmed.2026.1818918)
Supplement: Supplementary file 2 [file Table_1.docx]

| **Supplementary Table 1** |  |  |  |
| --- | --- | --- | --- |
| No. | Outcome measure | Domain | Definition / description |
| 1 | Pathological efficacy | Physical and chemical examination-Pathological examination | Proportion of patients showing histopathological improvement (e.g., reduced degree of atrophy or intestinal metaplasia) based on a standardised gastric biopsy scoring system (e.g., Updated Sydney System). |
| 2 | Efficacy of intestinal metaplasia treatment, pathological grading | Physical and chemical examination-Pathological examination | Proportion of patients with histopathological regression or resolution of intestinal metaplasia after treatment, graded according to a validated pathological classification. |
| 3 | Therapeutic efficacy for glandular atrophy | Physical and chemical examination-Pathological examination | Proportion of patients showing histopathological reduction in the extent or grade of gastric glandular atrophy. |
| 4 | Glandular Atrophy Score | Physical and chemical examination-Pathological examination | Numerical score (e.g., 0–3) quantifying the severity of gastric glandular loss, based on predefined pathological criteria (e.g., Updated Sydney System). |
| 5 | Intestinal metaplasia score | Physical and chemical examination-Pathological examination | Semi‑quantitative score (e.g., 0–3) reflecting the extent and severity of intestinal metaplasia in gastric mucosa on histopathological examination. |
| 6 | Atypical Hyperplasia Score | Physical and chemical examination-Pathological examination | Histological score grading the degree of gastric epithelial dysplasia (atypical hyperplasia), usually from mild (grade 1) to severe (grade 3). |
| 7 | Chronic Inflammation Score | Physical and chemical examination-Pathological examination | Score quantifying the density of chronic inflammatory cell (mainly lymphocytes and plasma cells) infiltration in gastric mucosa, typically 0–3. |
| 8 | Activity score | Physical and chemical examination-Pathological examination | Score representing the degree of acute/active inflammation (neutrophil infiltration) in the gastric mucosa, rated on a 0–3 scale. |
| 9 | Pathological Grading of Intestinal Metaplasia | Physical and chemical examination-Pathological examination | Categorical grade (e.g., mild/moderate/severe or type I/II/III) assigned to intestinal metaplasia based on histopathological evaluation. |
| 10 | Pathological Grading of Atrophy | Physical and chemical examination-Pathological examination | Categorical grade reflecting the severity of gastric glandular atrophy on histopathological examination, often using a 0–3 scale. |
| 11 | Pathological Grading of Dysplasia | Physical and chemical examination-Pathological examination | Categorical grade (e.g., low‑grade dysplasia, high‑grade dysplasia) of gastric epithelial neoplasia based on histopathological criteria. |
| 12 | Gastroscopy Grading, OLGA/OLGIM Scoring and Reversal Rate | Physical and chemical examination-Pathological examination | Endoscopic staging according to OLGA (atrophy) or OLGIM (intestinal metaplasia) systems; reversal rate is the proportion of patients showing stage reduction after treatment. |
| 13 | Pathological grading | Physical and chemical examination-Pathological examination | Generic histological grading of gastritis features (atrophy, intestinal metaplasia, inflammation) as per standardised classification (e.g., Updated Sydney System). |
| 14 | Pepsinogen I (PG I) | Physical and chemical examination-Gastric function indicators | Serum level of pepsinogen I measured by enzyme‑linked immunosorbent assay (ELISA); reflects the secretory function of gastric fundus and body chief cells. |
| 15 | Pepsinogen II (PG Ⅱ) | Physical and chemical examination-Gastric function indicators | Serum level of pepsinogen II measured by ELISA; reflects the overall status of gastric mucosa, including both fundic and antral glands. |
| 16 | PGR (PG Ⅰ/PG Ⅱ) | Physical and chemical examination-Gastric function indicators | Ratio of serum pepsinogen I to pepsinogen II; a reduced ratio (<3) suggests gastric corpus atrophy. |
| 17 | Gastrin (GAS) | Physical and chemical examination-Gastric function indicators | Serum gastrin‑17 level measured by ELISA or radioimmunoassay; indicates G‑cell function and antral status; often elevated in gastric atrophy. |
| 18 | Motilin (MLT) | Physical and chemical examination-Gastric function indicators | Plasma motilin level measured by radioimmunoassay; a gastrointestinal hormone that regulates gastric motility and interdigestive migrating motor complex. |
| 19 | Somatostatin (SS) | Physical and chemical examination-Gastric function indicators | Plasma or tissue level of somatostatin measured by ELISA/radioimmunoassay; an inhibitory hormone that suppresses gastric acid secretion and gastrin release. |
| 20 | Vasoactive intestinal peptide (VIP) | Physical and chemical examination-Gastric function indicators | Plasma or tissue VIP level measured by ELISA; a neuropeptide that relaxes gastric smooth muscle and regulates mucosal blood flow. |
| 21 | Tumour Necrosis Factor-α (TNF-α) | Physical and chemical examination-Inflammatory indicators | Serum or tissue level of TNF‑α measured by ELISA; a pro‑inflammatory cytokine involved in chronic gastritis and mucosal injury. |
| 22 | Interleukin-6 (IL-6) | Physical and chemical examination-Inflammatory indicators | Serum or tissue level of IL‑6 measured by ELISA; a pleiotropic cytokine with both pro‑inflammatory and regenerative functions in gastric mucosa. |
| 23 | Interleukin-11 (IL-11) | Physical and chemical examination-Inflammatory indicators | Serum or tissue level of IL‑11 measured by ELISA; a cytokine involved in mucosal repair and platelet production, also with inflammatory modulatory effects. |
| 24 | Interleukin-8 (IL-8) | Physical and chemical examination-Inflammatory indicators | Serum or tissue level of IL‑8 measured by ELISA; a CXC chemokine that mediates neutrophil recruitment and activation in gastric inflammation. |
| 25 | Interleukin-17 (IL-17) | Physical and chemical examination-Inflammatory indicators | Serum or tissue level of IL‑17 measured by ELISA; reflects Th17‑mediated inflammatory response, implicated in autoimmune and chronic inflammatory conditions. |
| 26 | Interleukin-10 (IL-10) | Physical and chemical examination-Inflammatory indicators | Serum or tissue level of IL‑10 measured by ELISA; an anti‑inflammatory cytokine that down‑regulates Th1 responses and limits tissue damage. |
| 27 | Neutrophil/lymphocyte ratio (NLR) | Physical and chemical examination-Inflammatory indicators | Ratio of absolute neutrophil count to absolute lymphocyte count in peripheral blood; a systemic inflammatory marker. |
| 28 | Interleukin-1β (IL-1β) | Physical and chemical examination-Inflammatory indicators | Serum or tissue level of IL‑1β measured by ELISA; a key pro‑inflammatory cytokine that activates immune cells and promotes gastric inflammation. |
| 29 | CD4+, CD8+, CD4+/CD8+ | Physical and chemical examination-Immune function indicators | Lymphocyte subset counts (CD4+ helper T cells, CD8+ cytotoxic T cells) and their ratio, measured by flow cytometry; reflects cellular immune status. |
| 30 | CEA, CDX2, Villin and Ki67 | Physical and chemical examination-Immunohistochemical indicators | Expression levels of carcinoembryonic antigen (CEA), intestinal transcription factor CDX2, enterocyte marker Villin, and proliferation marker Ki67 detected by immunohistochemistry; used to assess dysplasia and cancer risk. |
| 31 | GST-π, COX-1 and VR | Physical and chemical examination-Immunohistochemical indicators | Immunohistochemical expression of glutathione S‑transferase pi (GST‑π), cyclooxygenase‑1 (COX‑1), and vanilloid receptor (VR); involved in detoxification, mucosal protection, and pain perception. |
| 32 | Wnt3/β-catenin | Physical and chemical examination-Immunohistochemical indicators | Protein expression (by immunohistochemistry or Western blot) of Wnt3 and β‑catenin; reflects activation of the Wnt signaling pathway, which is implicated in gastric carcinogenesis. |
| 33 | Epidermal Growth Factor (EGF) | Physical and chemical examination-Vascular endothelial function indicators | Serum or tissue level of EGF measured by ELISA; a growth factor that promotes gastric epithelial cell proliferation and mucosal repair. |
| 34 | Vascular endothelial growth factor (VEGF) | Physical and chemical examination-Vascular endothelial function indicators | Serum or tissue level of VEGF measured by ELISA; a key inducer of angiogenesis, often increased in chronic inflammation and gastric lesions. |
| 35 | Human basic fibroblast growth factor (bFGF) | Physical and chemical examination-Vascular endothelial function indicators | Serum or tissue level of bFGF measured by ELISA; a growth factor that stimulates angiogenesis, fibroblast proliferation, and tissue repair. |
| 36 | HP eradication rate/HP positivity rate | Physical and chemical examination-HP detection | HP eradication rate = proportion of patients who become H. pylori negative after treatment (confirmed by a valid test); HP positivity rate = proportion of patients testing positive at a given time point. |
| 37 | Gastroscopy efficacy | Physical and chemical examination-Gastroscopy | Proportion of patients showing endoscopic improvement in gastric mucosal appearance (e.g., reduced erythema, oedema, atrophy, or nodules) based on standardised criteria. |
| 38 | Gastric mucosal scoring during gastroscopy | Physical and chemical examination-Gastroscopy | Endoscopic semi‑quantitative score (e.g., Kimura‑Takemoto classification or modified endoscopic grading) assessing the severity of gastric mucosal atrophy and intestinal metaplasia. |
| 39 | Gastroscopic imaging changes | Physical and chemical examination-Gastroscopy | Qualitative description of changes in endoscopic findings before and after treatment, such as improved vascular pattern, disappearance of mucosal nodules, or reduction of erosion. |
| 40 | Malondialdehyde (MDA) | Physical and chemical examination-Oxidative stress indicators | Serum or tissue level of malondialdehyde measured by thiobarbituric acid reactive substances (TBARS) assay; a marker of lipid peroxidation and oxidative stress. |
| 41 | Superoxide dismutase (SOD) | Physical and chemical examination-Oxidative stress indicators | Serum or tissue activity level of superoxide dismutase measured by enzymatic assay; an antioxidant enzyme that scavenges superoxide radicals. |
| 42 | Glutathione peroxidase (GSH-Px) | Physical and chemical examination-Oxidative stress indicators | Serum or tissue activity of glutathione peroxidase measured by enzymatic assay; an important antioxidant enzyme that reduces lipid hydroperoxides. |
| 43 | Serum TGF-α, CXCL10 | Physical and chemical examination-Other indicators | Serum levels of transforming growth factor‑alpha (TGF‑α) and chemokine CXCL10 measured by ELISA; involved in mucosal inflammation, immune regulation, and epithelial repair. |
| 44 | Syndrome Scoring System/Symptom Scoring System | TCM syndromes-TCM Syndrome Scoring | Total score based on a validated questionnaire assessing the severity of TCM syndrome patterns (e.g., spleen‑stomach deficiency) or individual symptoms (e.g., epigastric pain, bloating) before and after treatment. |
| 45 | Therapeutic Efficacy of TCM Syndromes | TCM syndromes-Therapeutic Efficacy of TCM in Treating Diseases and Syndromes | Proportion of patients achieving a predefined reduction in TCM syndrome score (e.g., ≥70% reduction) after treatment, indicating syndrome improvement. |
| 46 | Therapeutic Effects of TCM Symptoms | TCM syndromes-Therapeutic Efficacy of TCM in Treating Diseases and Syndromes | Proportion of patients with clinically significant improvement in individual TCM symptom scores (e.g., ≥50% reduction) after intervention. |
| 47 | Overall efficiency | Symptoms and signs-Overall efficiency | Composite outcome measure, usually based on a combination of symptom improvement, endoscopic findings, and/or pathological changes; definition varies across studies. |
| 48 | VAS | Symptoms and signs-Rating | A 10cm or 100mm line where patients rate the intensity of abdominal pain or discomfort (0 = no pain, 10/100 = worst possible pain). |
| 49 | Frequency of stomach pain episodes | Symptoms and signs-Rating | Number of stomach pain episodes recorded over a defined period (e.g., per week or per month), as reported by the patient or recorded in a diary. |
| 50 | Clinical Symptom Score | Symptoms and signs-Rating | Summed score of multiple gastritis‑related symptoms (e.g., pain, bloating, nausea, early satiety, acid reflux) evaluated by a standardised scale. |
| 51 | PRO Scale | Symptoms and signs-Rating | Patient‑Reported Outcome scale measuring symptom burden, functional status, and impact on daily life, using a validated instrument (e.g., Gastrointestinal Symptom Rating Scale). |
| 52 | HAMA Scale | Symptoms and signs-Rating | Hamilton Anxiety Rating Scale score; a clinician‑rated scale assessing the severity of anxiety symptoms, which may be associated with functional gastrointestinal disorders. |
| 53 | Time taken for abdominal pain and bloating to subside | Symptoms and signs-Rating | Time interval (e.g., days or weeks) between treatment initiation and complete resolution of abdominal pain and bloating as reported by the patient. |
| 54 | HP eradication rate, recurrence rate | Long-term prognosis | HP eradication rate at long‑term follow‑up (proportion of patients remaining H. pylori negative) and recurrence rate of atrophy/intestinal metaplasia or reinfection rate after treatment cessation. |
| 55 | SF-36 Health Survey Score | Quality of life | Short Form‑36 (SF‑36) health survey score measuring health‑related quality of life across eight domains (physical functioning, role physical, bodily pain, general health, vitality, social functioning, role emotional, mental health). |
| 56 | Economic evaluation | Length of hospital stay | Total number of days a patient remains hospitalised during the study period, used as a proxy indicator for direct medical cost burden. |
| 57 | Security incident | Security incident | Any adverse event or untoward medical occurrence (including intervention‑related and unrelated events) that occurs during the study period, regardless of causality. |
